# Supplementary figures and images for: Zwitterionic molecularly imprinted polymers for selective capillary microextraction of N1,N12-Diacetylspermine (DiAcSpm) from breast cancer
Source: PLoS One. 2026 Jan 20;21(1):e0339776. doi: 10.1371/journal.pone.0339776 (PMC12818627; doi:10.1371/journal.pone.0339776)

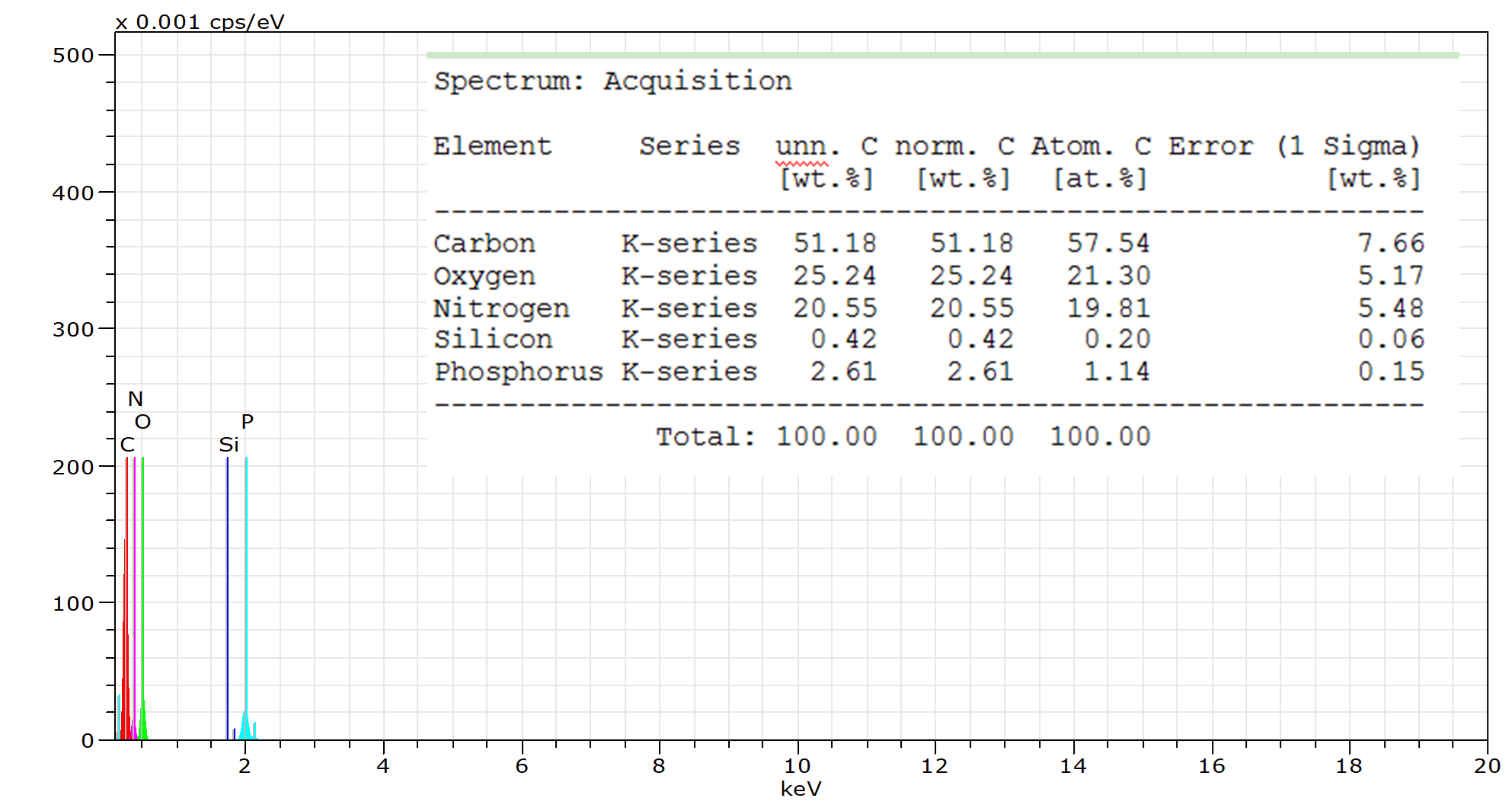

Supplement: S1 Fig — (TIF) [file pone.0339776.s001.tif]

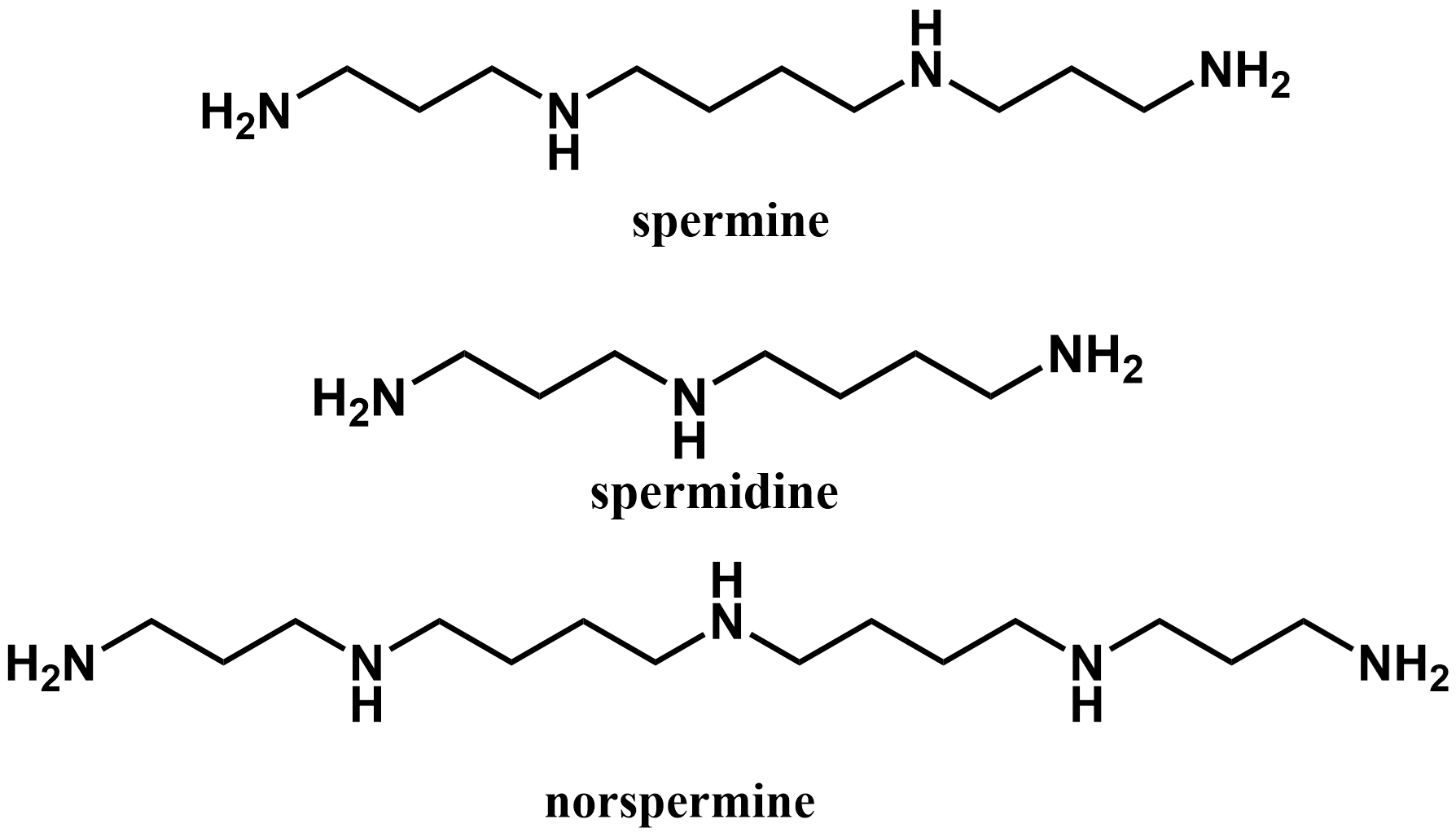

Supplement: S2 Fig — (PNG) [file pone.0339776.s002.png]

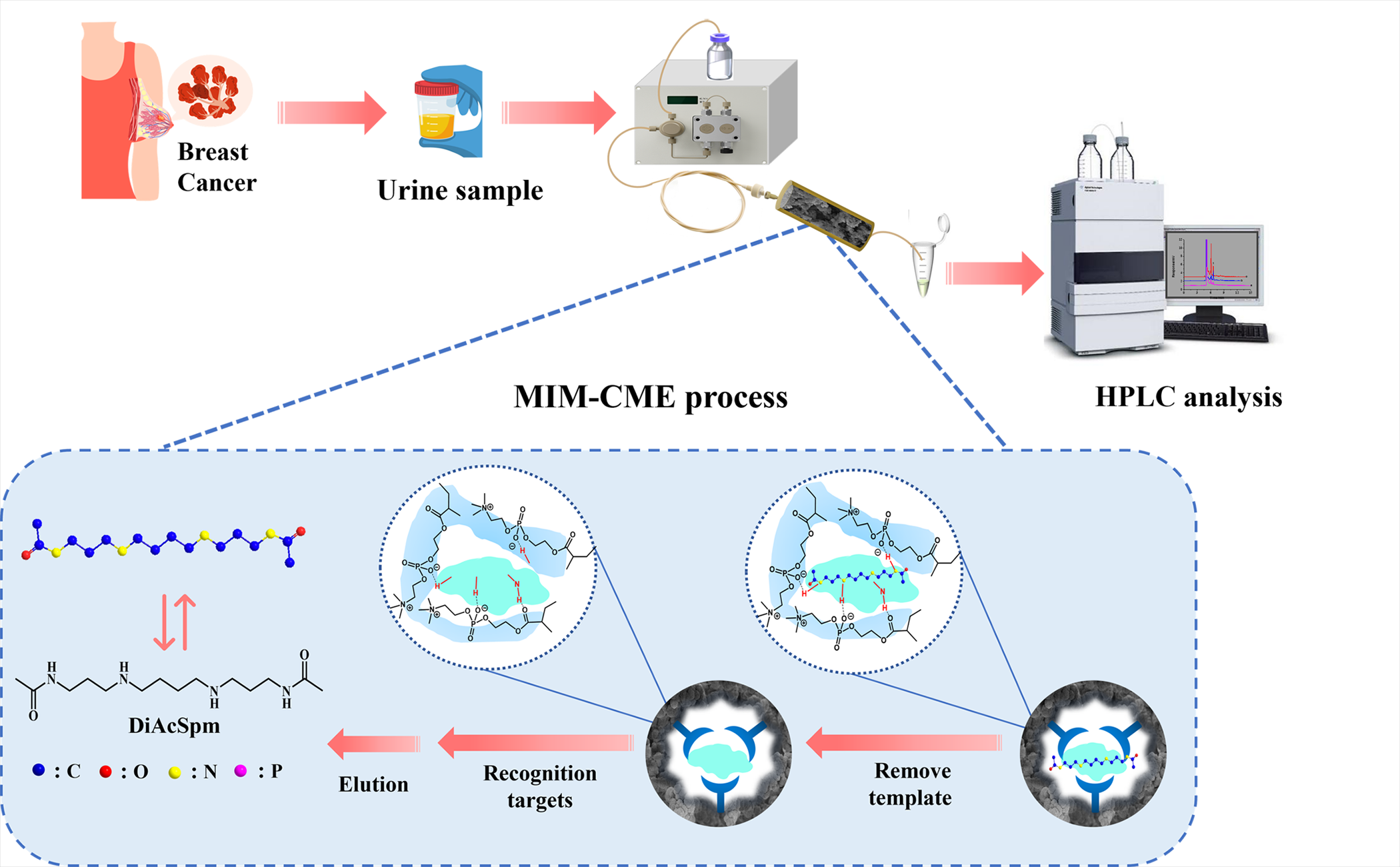

Supplement: S1 File — (TIF) [file pone.0339776.s006.tif]
