# Supplementary material for: Zwitterionic molecularly imprinted polymers for selective capillary microextraction of N1,N12-Diacetylspermine (DiAcSpm) from breast cancer
Source: PLoS One. 2026 Jan 20;21(1):e0339776. doi: 10.1371/journal.pone.0339776 (PMC12818627; doi:10.1371/journal.pone.0339776)
Supplement: S3 Fig — Peak: 1: DiAcSpm. (DOCX) [file pone.0339776.s003.docx]

**Fig S3. The HPLC-UV chromatograms of (a) blank sample; 25 μM standard DiAcSpm sample on (b) directly injection; (c) extraction with NIM column and (c) extraction with MIM column. Peak: 1: DiAcSpm.**
