# Supplementary material for: Zwitterionic molecularly imprinted polymers for selective capillary microextraction of N1,N12-Diacetylspermine (DiAcSpm) from breast cancer
Source: PLoS One. 2026 Jan 20;21(1):e0339776. doi: 10.1371/journal.pone.0339776 (PMC12818627; doi:10.1371/journal.pone.0339776)
Supplement: S1 Table — (DOCX) [file pone.0339776.s004.docx]

**Table S1. Quantitative evaluation of the MIM selectivity.**

|  | **Analyte** | **Structure** | **IF^a^** | **k^b^** |
| --- | --- | --- | --- | --- |
|  | DiAcSpm | C₁₈H₃₈N₄O₂ | 3.5 | / |
|  | Spermine | C₁₀H₂₆N₄ | 1.8 | 1.94 |
|  | Spermidine | C₇H₁₉N₃ | 1.3 | 2.69 |
|  | Norspermine | C₁₁H₂₈N₄ | 1.5 | 2.33 |

^a^ Imprinting factor (IF): IF = Q_MIM_ / Q_NIM_, where Q is the adsorption capacity.

^b^ Selectivity coefficient (k): k = IF_DiAcSpm_ / IF_competitor_.
