# Supplementary material for: Zwitterionic molecularly imprinted polymers for selective capillary microextraction of N1,N12-Diacetylspermine (DiAcSpm) from breast cancer
Source: PLoS One. 2026 Jan 20;21(1):e0339776. doi: 10.1371/journal.pone.0339776 (PMC12818627; doi:10.1371/journal.pone.0339776)
Supplement: S2 Table — (DOCX) [file pone.0339776.s005.docx]

**Table S2.** **Comparative evaluation of qnalytical methods for polyamine quantification.**

| Analytes | Matrix / Sample Type | Detection Technique | LOD (μM) | Recovery (%) | Run Time (min) | Derivatization | References |
| --- | --- | --- | --- | --- | --- | --- | --- |
| 9 PAs & 8 Steroids | Human Serum | LC-MS/MS | ~0.006 – 0.06 | 87.8 – 123.6 | 23 (Incl. deriv.) | Yes (for PAs) | [1] |
| PUT, SPM | Pleural Effusion Cells | HPLC-HRMS (SWATH) | ~0.01 – 0.05 | 90.95 – 102.77 | 3.0 (Chromatography) | No | [2] |
| CAD, PUT, SPD, SPM | Standard Solution | ERLIC-MS/MS | 0.04 – 0.99 | N.R. | 10 | No | [3] |
| PUT, SPD, SPM | Poultry Tissue | HPLC-UV | 0.14 – 0.37 | 94.73 – 106.58 | 9 | Yes | [4] |
| DiAcSpm | Clinical Samples | MIM-CME-HPLC-UV | 3.3 | 76.8% -91.2% | 8 | No | This work |

AA: Amino Acid; CAD: Cadaverine; DiAcSpm: Diacetylspermine; LOD: Limit of Detection; LOQ: Limit of Quantification; MIM: Molecularly Imprinted Polymer; N.R.: Not Reported; PA: Polyamine; PUT: Putrescine; RSD: Relative Standard Deviation; SPD: Spermidine; SPM: Spermine.

**References**

1. Lee YR, Lee JW, Hong J, Chung BC (2021) Simultaneous Determination of Polyamines and Steroids in Human Serum from Breast Cancer Patients Using Liquid Chromatography–Tandem Mass Spectrometry. Molecules 26:1153
2. Wang T, Pan Y, Hu X, Ren W, Li L, Huang J (2024) Simultaneous detection of 10 cancer-associated amino acids and polyamines by high-performance liquid chromatography-high resolution mass spectrometry in pleural effusion cells obtained from lung adenocarcinoma patients. J Pharm Biomed Anal 251:1164
3. Dörfel D, Rohn S, Jantzen E (2024) Electrostatic Repulsion Hydrophilic Interaction Liquid Chromatography (ERLIC) for the Quantitative Analysis of Polyamines. J Chromatogr A 1720:464820
4. Jiang D, Ling W, Yi Z, Jiang Y, Wang Z, An X, Ji C, Wang X, Sun Q, Kang B (2024) High-performance liquid chromatography determination of polyamine contents in poultry based on precolumn benzoyl chloride derivatization. Microchemical Journal 203:110821
