# Supplementary material for: Zwitterionic molecularly imprinted polymers for selective capillary microextraction of N1,N12-Diacetylspermine (DiAcSpm) from breast cancer
Source: PLoS One. 2026 Jan 20;21(1):e0339776. doi: 10.1371/journal.pone.0339776 (PMC12818627; doi:10.1371/journal.pone.0339776)
Supplement: Attachment — Submitted filename: PONE.docx [file pone.0339776.s007.docx]

ID: PONE-D-25-32785

Title: Zwitterionic Molecularly Imprinted Polymers for Selective Capillary Microextraction of Spermine from Breast Cancer

This manuscript is suitable for publication in this journal after major revisions.

1. How stable is the MIM column over multiple extraction cycles? Can you provide a column reusability study?

2. Add statistical analysis (e.g., t-test or ANOVA) to compare MIM and NIM performance with significance levels.

3. Clearly define the recovery calculation formula and add confidence intervals to recovery and precision data.

4. Discuss clinical relevance of the LOD/LOQ values in the context of known spermine/DiAcSpm concentrations in patient populations.

5. Incorporate a visual workflow figure (graphical abstract) summarizing the CME process and advantages.

6. Expand on the novelty of your work—how is your approach substantially different from previous MIP or zwitterionic-MIP studies?

7. Compare your results (LOD, linear range, recovery) with other published methods in a concise benchmarking table.

8. Clarify whether the optimized extraction parameters were validated across multiple MIM batches.

9. How does the binding affinity of the MIM column vary with pH or ionic strength?

10. Improve figure readability (especially SEM and BET plots) by labeling axes clearly and adding scale bars and annotations.

11. Were matrix effects quantitatively evaluated (e.g., via matrix-matched calibration or recovery experiments)?
