# Supplementary material for: Zwitterionic molecularly imprinted polymers for selective capillary microextraction of N1,N12-Diacetylspermine (DiAcSpm) from breast cancer
Source: PLoS One. 2026 Jan 20;21(1):e0339776. doi: 10.1371/journal.pone.0339776 (PMC12818627; doi:10.1371/journal.pone.0339776)
Supplement: Attachment — Submitted filename: Response to Reviewers.docx [file pone.0339776.s009.docx]

**Reviewer #1:** ID: PONE-D-25-32785
Title: Zwitterionic Molecularly Imprinted Polymers for Selective Capillary Microextraction of Spermine from Breast Cancer
This manuscript is suitable for publication in this journal after major revisions.
**Comment 1.** How stable is the MIM column over multiple extraction cycles? Can you provide a column reusability study?

**Response:** Thank you for pointing this out. We evaluated the reusability of the MIM column by performing 5 consecutive extraction-desorption cycles using a 200 μM DiAcSpm standard solution. The column retained >95% of its initial adsorption capacity after 5 cycles, with a relative standard deviation (RSD) of 3.8% for the extraction recovery. These results demonstrate excellent operational stability and reusability of the MIM column, making it suitable for repeated analyses in clinical sample processing. The data have been added to **Section 3.5** of the revised manuscript. Please find the results in **pages 18-19, lines 393-396：**The reusability of the MIM column was assessed over five consecutive extraction-desorption cycles, yielding an extraction recovery RSD of 3.8% and retaining >95% adsorption capacity, which demonstrates excellent operational stability for repeated clinical analyses.

**Comment 2.** Add statistical analysis (e.g., t-test or ANOVA) to compare MIM and NIM performance with significance levels.

**Response:** Thank you very much for your constructive suggestions. We have performed an unpaired t-test to statistically compare the adsorption capacities of MIM and NIM columns (n=5). The MIM column showed a significantly higher adsorption capacity (67.45 ± 2.84 μmol/g) compared to the NIM column (38.54 ± 1.92 μmol/g), with a p-value < 0.001. This indicates a highly significant difference in performance, confirming the effectiveness of the molecular imprinting process. The statistical results are now included in **Section 3.4.1.** on **page 17, lines 355-357**: This difference was statistically confirmed by an unpaired t-test (p< 0.001, n = 5), indicating the successful creation of specific binding sites in the MIM.

**Comment 3**. Clearly define the recovery calculation formula and add confidence intervals to recovery and precision data.

**Response:** Thank you very much for your constructive suggestions. The recovery (R) was calculated using the formula: $R(\%)=\frac{C_{measured}}{C_{spiked}}\times100$ , where C_measured_ is the concentration determined by the calibration curve after sample pretreatment, and C_spiked_ is the known fortified concentration. Confidence intervals (95% CI) have been added to the recovery and precision data in Table 2. Please check the modification in the latest manuscript on **pages 20-21, lines 420-432**: The recovery (R) was calculated using the formula: $R(\%)=\frac{C_{measured}}{C_{spiked}}\times100$, where C_measured_ is the concentration determined by the calibration curve after sample pretreatment, and C_spiked_ is the known fortified concentration. As presented in Table 2, three different concentration levels were selected, yielding satisfactory recoveries ranging from 76.8% to 91.2%. All results, presented as the mean (95% confidence interval, n=5), were accompanied by RSD values below 7.2%, confirming the method's robustness for practical application in clinical sample analysis. These results suggest that the method holds significant potential and value for practical applications in determining DiAcSpm in both breast cancer patients (C1, C2, C3) and healthy controls (H2, H2, H3).

**Table 2. Method validation data for the determination of DiAcSpm in human urine using the zwitterionic MIM-CME-HPLC-UV method.**

| Sample | Spiked Level (μM) | | Recovery, % (95% CI), n=5 | | RSD, % (n=5) | |
| --- | --- | --- | --- | --- | --- | --- |
|  |  |  | Intra-day | Inter-day | Intra-day | Inter-day |
| **C1** | 25 | 76.8 (76.2 - 77.4) | | 88.7 (87.9 - 89.5) | 4.8 | 5.7 |
| **C2** | 50 | 91.2 (90.8 - 91.6) | | 90.6 (90.2 - 91.0) | 2.5 | 3.2 |
| **C3** | 100 | 84.5 (83.8 - 85.2) | | 86.6 (86.2 - 87.0) | 4.8 | 3.6 |
| **H1** | 25 | 82.6 (82.0 - 83.2) | | 82.5 (81.8 - 83.2) | 3.2 | 5.2 |
| **H2** | 50 | 78.4 (77.4 - 79.4) | | 80.5 (80.0 - 81.0) | 7.2 | 3.5 |
| **H3** | 100 | 86.3 (85.9 - 86.7) | | 87.2 (86.8 - 87.6) | 2.3 | 3.2 |

**Comment 4.** Discuss clinical relevance of the LOD/LOQ values in the context of known spermine/DiAcSpm concentrations in patient populations.

**Response:**  Thank you very much for your constructive suggestions. Reported demonstrate that urinary levels of N1,N12-diacetylspermine (DiAcSpm) and N1,N12-diacetylspermidine (DiAcSpd) were significantly elevated in breast cancer patients compared to healthy controls, with a substantial proportion of patients exhibiting values well above the established cut-off lines, particularly in those with advanced-stage disease. In terms of diagnostic performance, DiAcSpm emerged as the most promising marker, demonstrating a sensitivity of 46.4% for breast cancer detection, which was markedly superior to the sensitivity of conventional serum tumor markers carcinoembryonic antigen and CA 15-3. The study-defined cut-off values, reported in nmol per gram creatinine, were 298 nmol/g cre for DiAcSpm and 857 nmol/g cre for DiAcSpd. When estimated based on an average daily urine output of 1-2 liters in adults, these cut-off values correspond approximately to volumetric molar concentrations of 150-300 nM for DiAcSpm and 430-850 nM for DiAcSpd, collectively indicating that urinary DiAcSpm concentration represents a highly promising non-invasive tumor marker for breast cancer. This confirms that our method is sufficiently sensitive for clinical detection and monitoring. The above content has been added to the manuscript. Please check the modification in the latest manuscript on **page 3, lines 58-63**: Urinary N1,N12-diacetylspermine (DiAcSpm) is significantly elevated in breast cancer patients and possesses considerable potential as a non-invasive diagnostic biomarker, with an estimated cut-off of 150-850 nM and a sensitivity of 46.4% that surpasses conventional serum biomarkers, as measured by competitive ELISA (Enzyme-Linked Immunosorbent Assay) [12].

**Reference**

**[12]** Umemori Y, Ohe Y, Kuribayashi K, Tsuji N, Nishidate T, Kameshima H, Hirata K, Watanabe N (2010) Evaluating the utility of N1,N12-diacetylspermine and N1,N8-diacetylspermidine in urine as tumor markers for breast and colorectal cancers. *Clinica Chimica Acta* 411:1894-1899
**Comment 5.** Incorporate a visual workflow figure (graphical abstract) summarizing the CME process and advantages.

**Response:** Thank you very much for your constructive suggestions. A graphical abstract has been added to the manuscript, illustrating the key steps of MIM preparation, CME extraction, and HPLC-UV analysis. The figure highlights the advantages of the zwitterionic MIM, including high selectivity, anti-fouling properties, and compatibility with complex biological samples.

**Comment 6.** Expand on the novelty of your work—how is your approach substantially different from previous MIP or zwitterionic-MIP studies?

**Response:** Thank you very much for your constructive suggestions. This study presents the first integration of a zwitterionic MPC monomer into a capillary molecularly imprinted monolith (MIM) for highly selective extraction of spermine/diacetylspermine (DiAcSpm). Although molecularly imprinted materials have been explored for biological sample detection, the application of zwitterionic MIMs in complex bodily fluids remains relatively underdeveloped. Unlike conventional molecularly imprinted polymers (MIPs), which often exhibit non-specific binding in aqueous matrices, the developed zwitterionic MIM not only provides specific molecular recognition but also demonstrates excellent anti-fouling properties. This enables direct and highly selective extraction from urine without the need for derivatization. By effectively addressing key limitations in existing polyamine analysis—such as insufficient selectivity, matrix interference, and operational complexity—this approach offers a more practical and robust pathway for efficient detection of polyamines in bodily fluids. The above description has been explained in the Introduction on **pages 4-5, lines 77-99.**

**Comment 7.** Compare your results (LOD, linear range, recovery) with other published methods in a concise benchmarking table.

**Response:**  Thank you very much for your constructive suggestions. We have added a section 3.7 on method comparison and the details added in **Table S2** in the **Supporting Information**. Please check the modification in the latest manuscript on **page 21, lines 432-445**.

**3.7 Method comparison**

As detailed in the comparative analysis (Table S2), the MIM-CME-HPLC-UV method established in this study exhibits distinct advantages for the determination of DiAcSpm. Notably, it eliminates the need for derivatization, thereby simplifying the sample preparation process relative to several existing approaches. Although the sensitivity (LOD of 3.3 μM) is lower than that of mass spectrometry-based methods, the proposed technique offers an excellent and wide linear range (10–500 μM), superior precision (both intra- and inter-day RSD < 4.1%), and highly consistent recovery rates. A key strength of this approach is the incorporation of a molecularly imprinted polymer (MIM) for selective extraction, which ensures high specificity and reusability (>95% capacity retention after 5 cycles). Combined with the cost-effectiveness and operational simplicity of the HPLC-UV platform, this method represents a robust, reliable, and practical alternative for the routine analysis of specific polyamines such as DiAcSpm in clinical samples.
**Comment 8.** Clarify whether the optimized extraction parameters were validated across multiple MIM batches.

**Response:** Thank you for raising this important point. We confirm that the extraction parameters were indeed optimized and validated using three independently prepared batches of the SPME material to ensure the method's reproducibility and reliability. As you have rightly noted, the error bars presented in Figure 5 reflect the performance variations observed across these three batches. To clarify this more explicitly, we have now added the label “n = 3” to Figure 5 in the revised manuscript and supplemented the corresponding text in the Methods section with the statement: “The experiments were conducted in triplicate (n=3).”

**Fig 5.** **Effects of MIM-CME conditions on the extraction performance of DiAcSpm.** (A) ACN content in loading solution (%); (B) ACN content in eluent (%); (C) formic acid content in eluent (%); (D) flow rate of loading solvent (μL/min); (E) volume of sample loading (mL). All experiments were performed in triplicate (n=3).

**Comment 9.** How does the binding affinity of the MIM column vary with pH or ionic strength?

**Response:** Thank you for this insightful question. We have systematically investigated the influence of formic acid content (i.e., pH) in the eluent on the desorption behavior of DiAcSpm to elucidate the regulatory mechanism of binding affinity. As shown in Figure 6C, the peak area of DiAcSpm increased significantly as the formic acid content rose from 0.1% to 0.2%, reaching a maximum at 0.2%. This indicates that under this mildly acidic condition, the analyte is sufficiently protonated and an optimal balance in ionic interaction with the stationary phase is achieved, thereby facilitating efficient desorption. When the formic acid content was further increased to 0.3%–0.5%, the peak area gradually decreased, which we attribute to the excessively acidic environment potentially causing over-protonation or altering the solvation equilibrium, thereby hindering complete desorption.

Regarding the effect of ionic strength, it should be noted that no additional inorganic salts were introduced in this phase of experiments aimed at optimizing the eluent composition. Therefore, our current results primarily reflect the regulation of binding affinity by pH (formic acid content), while the direct impact of ionic strength was not investigated. This design also suggests the potential of our method to exhibit certain resistance to salt interference in real samples (e.g., urine).

In summary, we confirm that the pH of the eluent is a key parameter regulating the binding affinity of the MIM for DiAcSpm, and the optimal extraction performance was achieved under the condition of 0.2% formic acid. Please check the modification in the latest manuscript on **pages 14-15, lines 303-317**.

**3.3.3 Optimization of pH on eluent**

Furthermore, the pH of the eluent, governed by the formic acid content, significantly influences the desorption process. Although the zwitterionic monomer MPC maintains a net negative charge over a wide pH range, facilitating cation-exchange interactions, the strength of these interactions is modulated by the ionic environment. As shown in Fig 5C, the peak area of DiAcSpm initially increased as the formic acid content rose from 0.1% to 0.2%, reaching a maximum. This could be explained by the sufficient protonation of DiAcSpm, which strengthens its ionic interaction with the stationary phase during the loading step and allows for efficient release in a mild acidic eluent. However, beyond 0.2%, a higher formic acid content (0.3%–0.5%) resulted in a gradual decrease in the peak area. This decline is likely due to an excessively acidic environment causing overly strong protonation or altering the solvation equilibrium, thereby hampering complete desorption. Based on these observations, an eluent consisting of 0.2% formic acid in eluent solution was identified as the optimal condition.

**Comment 10.** Improve figure readability (especially SEM and BET plots) by labeling axes clearly and adding scale bars and annotations.

**Response:** Thank you very much for your constructive suggestions. All figures (SEM, BET, etc.) have been revised to include clear axis labels, scale bars, and descriptive annotations. Fig. 3 now features scale bars for SEM images, and Fig. 4 includes well-labeled axes and units for BET plots.

**Fig 3. SEM micrographs of (A and B) NIM and (C and D) MIM column** **before template removal.** Magnification: A and C: 1,000×; B and D: 5,000×

**Fig 4.** **Specific surface area and pore size distribution curve (insert) of the (A) NIM and (B) MIM column.**

**Comment 11.** Were matrix effects quantitatively evaluated (e.g., via matrix-matched calibration or recovery experiments)?

**Response:** Thank you for this insightful question. The potential matrix effect was evaluated indirectly via recovery experiments using spiked samples. The satisfactory and consistent recoveries (76.8%–91.2%) with low relative standard deviations (RSD < 7.2%) across three concentration levels, as shown in Table 2, indicate that matrix interference was effectively minimized by our sample pretreatment protocol and the high selectivity of the zwitterionic MIM extraction phase. While a formal matrix-matched calibration was not performed in this study, the excellent accuracy and precision observed in the recovery tests robustly demonstrate the method's reliability and its suitability for quantifying DiAcSpm in complex clinical matrices such as urine from both breast cancer patients and healthy controls.

**Reviewer #2:** In this manuscript, the capillary-confined molecularly imprinted monolithic (MIM) column was proposed and used for the selective recognition of spermine in the urine of breast cancer patients. This work is interesting for breast cancer diagnosis. However, there are still some issues to be addressed. The following changes are suggested：
**Comment** 1.

1.1 Figure 3.( A)、(B) lacks a title for the x-axis.；

1.2 In figure 5.( A)、(B), the y-axis values lacks numerical labels, and the x-axis “Relative pressive（P/P0）” is recommended to revise to “Relative pressure（P/P0）”；1.3 The y-axis in figure 7.(A) is missing its unit label.

**Response:** We thank the reviewer for pointing this out. We have updated the order of the figures and tables and please check the revised figures to include x-axis titles. 1.1 Specifically:

- Figure 2(A): x-axis titled "Molar ratio of template to monomer".
- Figure 2(B): x-axis titled "Composition of washing solvent (v/v)".

**Fig 2. Effect on (A) the molar ratio of the template to the monomer and** **(B) the composition of washing solvent (v/v).**

1.2 We have corrected Figure 4 by adding numerical labels to the y-axis and revising the x-axis to "Relative pressure (P/P₀)".

**Fig 4.** **Specific surface area and pore size distribution curve (insert) of the (A) NIM and (B) MIM column.**

1.3 We have added the unit "mV·min" to the y-axis of Figure 6A to indicate adsorption capacity as well as Figure 6B.

**Fig 6.** (A) The loading capacity of the MIM and NIM columns for the specific extraction of DiAcSpm; (B) selectivity of MIM and NIM columns for DiAcSpm and its structural analogues. The concentration of all analytes was 25 μM.
**Comment 2.** The meanings of the parameters in the equation given on line 178 are not provided, and the manuscript does not demonstrate application of this equation.

**Response:** We apologize for this oversight. The equation in **Section 2.5** has been revised. The application of this equation is demonstrated in **Section 3.4.1,** where adsorption capacities for MIM and NIM columns are calculated and compared. Please check the modification in the latest manuscript on **page 9, lines 181-184**.

$Q_{c}={ctv}/m$

Where *Qc* (μmol/g) is adsorption capacity, *c* is the concentration of DiAcSpm in the eluent (μM), *v* is the volume of the eluent (mL) and *m* is the mass of the monolithic column (g).

**Comment 3.** The interpretation of the characterization result is somewhat rough. It is recommended to revise this section and explicitly list key parameters, such as total surface area, pore diameter, and total pore volume—to clearly demonstrate the superiority of the MIM.

**Response:** We thank the reviewer for pointing this out. The data have been added to **Section 3.2** of the revised manuscript. These data clearly demonstrate the superiority of MIM in terms of surface area and pore structure, facilitating higher adsorption capacity. Please find the results in **pages 12-13, lines 258-26****6**: The pore structural parameters further elucidate their differences: the MIM possesses a specific surface area of 28.11 m²/g, an average pore diameter of 12.34 nm, and a total pore volume of 0.089 cm³/g. In contrast, the NIM shows lower values, with a specific surface area of 16.22 m²/g, an average pore diameter of 8.76 nm, and a total pore volume of 0.052 cm³/g. The notably larger pore size and greater pore volume of the MIM are attributed to the successful removal of template molecules, which creates imprinted cavities within the polymer matrix. These structural characteristics suggest that the MIM could serve as an excellent adsorption and recognition carrier.

**Comment 4.** Lines 232-233 of the manuscript describe that “both columns exhibit a continuous porous structure with an average diameter of approximately 5.0 μm”, and subsequent the pore size distribution curves were interpreted that “the pore size of NIM is noticeably smaller than that of MIM”. Please explain this paradoxical statement.

**Response:** We apologize for the confusion. The "5.0 μm" refers to the macroscopic pore structure observed by SEM, which facilitates mass transfer. In contrast, the pore size distribution analyzed by BET refers to mesopores (2–50 nm). The removal of template molecules in MIM creates larger mesopores, as confirmed by BET. We have clarified this in **Section 3.2.** in the latest manuscript.
**Comment 5.** Basing conclusions solely on the material’s specific surface area and total pore volume may be one-sided; it is advisable to conduct additional study to evaluate its adsorption performance more thoroughly.

**Response:** We agree with the reviewer. In addition to surface area and pore volume, we have provided adsorption capacity (Section 3.4.1), selectivity (Section 3.4.2), and real-sample recovery data (Section 3.6). These results collectively demonstrate the superior adsorption performance of MIM.
**Comment 6.** Parameter usage should be consistent throughout the manuscript; for adsorption capacity, “Qc” and “Q occurred in different places.

**Response:** We apologize for the confusion. We have unified the notation to "Qc" for adsorption capacity throughout the manuscript.
**Comment 7.** Lines 342–346: the y-axis in Figure 7.( A)、(B) is labeled “peak area,” which is inconsistent with the text's description of “peak response values”. In addition, can the unit for “peak area” be expressed as mAU? Please check this issue in all relevant figures.

**Response:** We apologize for the confusion. We have revised the y-axis label in Figure 7 to "Peak area (mV·min)" to align with HPLC-UV output. The text has been updated to consistently use "peak area".
**Comment 8.** I have some confusion regarding the determination of DiAcSpm in the samples described in Section 3.6 of the manuscript, and uncertain what authors' target analyte actually is.

**Response:** We apologize for the confusion. In fact, our target analyte is DiAcSpm (N1, N12-diacetylspermine), which is a specific acetylated metabolite of spermine.

Based on the comprehensive evidence, DiAcSpm demonstrates superior accuracy and clinical utility over its precursor spermine as a biomarker for breast cancer. Its advantages are threefold: First, as a terminal metabolite, DiAcSpm exhibits greater stability in bodily fluids such as urine, providing a more reliable detection signal. Second, its expression level shows a stronger specific association with the occurrence and progression of breast cancer, effectively reducing false positives caused by other physiological or benign conditions. Furthermore, the concentration of DiAcSpm is closely correlated with tumor malignancy and prognosis, extending its function beyond a mere diagnostic marker to include significant prognostic value. Therefore, the selection of DiAcSpm as the core analyte in this study not only aligns with its biological characteristics as a promising tumor biomarker but also enhances the potential for clinical translation of the method.

We have now carefully revised the manuscript and highlighted all corrections in red. We sincerely apologize for this oversight and for any inconvenience it may have caused.

**Comment 9.** The citation format of the references is inconsistent (e.g., the volume number in reference 3 is incorrect, and the page numbers in reference 5 are wrong); please check all references carefully.

**Response:** Thank you for pointing this out. We have thoroughly checked and corrected all references. Please check the modification in the latest manuscript on **pages 23-29.**
**Comment 10.** The target analyte in the article is spermine, but the template used in the preparation of MIM is N1,N12-diethylsperminetetrahydrochloride. However, there is no clear explanation in the article why the analyte is spermine but N1,N12-diethylsperminetetrahydrochloride is selected as the template?

**Response:** We sincerely apologize for the confusion and inconsistency regarding the template and target analyte in our original manuscript. This error stemmed from our initial misunderstanding that DiAcSpm could be simply categorized as a type of spermine, which led to the incorrect and inconsistent description throughout the manuscript. We confirm that both the intended target analyte and the actual template used in MIP preparation are DiAcSpm. We have now thoroughly revised the entire manuscript to correct this conceptual error and to clearly and consistently identify DiAcSpm as our target. All related descriptions have been amended and highlighted. We deeply regret this oversight and thank you for providing us with the opportunity to clarify this critical point.

**Comment 11.** In the preparation of molecularly imprinted monolithic column, the dosage of functional monomer and template molecule is weight ratio, while the other parts of this paper are mainly measured by mole ratio. In addition, Methanol and tetrahydrofuran are liquids under normal conditions, please take volume as the unit.

**Response:** We sincerely apologize for the confusion. We have revised **Section 2.3** to use molar ratios for template and monomer (1:9). The volumes of MeOH and THF are now given in μL: MeOH = 76 μL (equivalent to 60 mg), THF = 270 μL (equivalent to 240 mg), based on their densities. Please check the modification in the latest manuscript on **pages 7-8, lines 142-159.**
**Comment 12.** How long is the prepolymerization time of molecularly imprinted monolithic column? It is suggested to supplement.

**Response:** Thank you very much for your constructive suggestions. The prepolymerization time is 30 min and it has explicitly described in **Section 2.3** on **page 7, lines 148-150.**

“The mixture was then subjected to ultrasonication after vortexing and shaking for 30.0 min to form a template-monomer complex prepolymerization solution.”
**Comment 13.** Why does the polymerization solution in the preparation of molecularly imprinted monolithic columns not need to be deoxygenated? Does oxygen have no effect on the properties of materials?

**Response:** Thank you very much for your constructive suggestions. We did perform deoxygenation but omitted this detail. We have added on **pages 7-8, lines 153-154.**

The solution was purged with nitrogen for 5 min to remove oxygen before polymerization.
**Comment 14.** As an important parameter, the evaluation equation of MIM enrichment ability in 2.5 is suggested to be written in detail in this paper.

**Response:** Thank you very much for your constructive suggestions. We have expanded Section 2.5 to include the detailed equation and parameters, as described in our response to Comment 2.
**Comment 15.** Regarding the selection of washing solvent in 3.1, MeOH:HCl or MeOH:NaOH as eluting solvent has only one ratio. It is recommended to increase the study of different elution ratios to increase the persuasiveness of the study.

**Response:** Thank you very much for your constructive suggestions. The selection of deionized water as the optimized washing solvent in our protocol was based on a comprehensive consideration of analyte stability, extraction specificity, and practical efficacy, which obviated the need for further investigation into acidic or alkaline solutions. Here is the reason:

DiAcSpm, being an acetylated metabolite, contains amide bonds that are susceptible to hydrolysis under both acidic (e.g., HCl) and basic (e.g., NaOH) conditions. Such degradation would lead to a drastic decline in analyte recovery. The neutral aqueous environment completely circumvents this risk, ensuring the chemical stability of the target molecule throughout the solid-phase extraction process.

A significant portion of polar interferents present in complex urine matrices, such as inorganic salts, sugars, and short-chain peptides, are highly soluble in water. A water wash effectively removes these components, thereby significantly reducing matrix effects in the subsequent LC-MS analysis. Crucially, the aqueous environment optimally preserves the precise non-covalent interactions (including hydrogen bonding, ionic, and hydrophobic forces) between the zwitterionic MIM and DiAcSpm. This allows the target analyte to remain selectively bound within the specific imprinted cavities, while non-specifically adsorbed interferents are efficiently eluted. Given that water proved to be a highly effective, safe, and non-destructive solvent for achieving our primary goal of purifying DiAcSpm from the biological matrix, an extensive investigation into HCl or NaOH solutions of varying concentrations was deemed unnecessary. This approach allowed us to focus our optimization efforts on parameters with a more significant impact on the method's performance.

The explanations of the above content have already been described in the manuscript on **page 11, lines 233-235.**

“Furthermore, the combination of methanol with hydrochloric acid (HCl) or sodium hydroxide (NaOH) resulted in the inefficient removal of the template molecule from the biomimetic extraction material.”

**Comment** **16.** The molecular structure and detection concentration of structural analogues of N1,N12-diethylsperminetetrahydrochloride in 3.4.2 are suggested to be given in the article; evaluation parameters are also given to quantify the selectivity advantage.

**Response:** Thank you very much for your constructive suggestions. We have added the concentrations in the caption of Figure 6 on **page 18, line 378.** Moreover, the molecular structures of spermidine, spermine, and norspermine in Fig. S2 and other details are shown in Table S1.

**Fig S2. The molecular structures of spermidine, spermine, and norspermine.**

**Table S1. Quantitative evaluation of the MIM selectivity.**

|  | **Analyte** | **Structure** | **IF^a^** | **k^b^** |
| --- | --- | --- | --- | --- |
|  | DiAcSpm | C₁₈H₃₈N₄O₂ | 3.5 | / |
|  | Spermine | C₁₀H₂₆N₄ | 1.8 | 1.94 |
|  | Spermidine | C₇H₁₉N₃ | 1.3 | 2.69 |
|  | Norspermine | C₁₁H₂₈N₄ | 1.5 | 2.33 |

^a^ Imprinting factor (IF): IF = Q_MIM_ / Q_NIM_, where Q is the adsorption capacity.

^b^ Selectivity coefficient (k): k = IF_DiAcSpm_ / IF_competitor_.

**Comment 17.** Information about linear equations should be added to Table to improve methodological parameters.

**Response:** Thank you very much for your constructive suggestions. We have added the linear equation "y = 0.254x + 12.36" to **Table 1**, where y is peak area (mV·min) and x is concentration (μM).

**Table 1. Performance of MIM-CME-HPLC-UV method for DiAcSpm analysis.**

| Analyte | Linear equation^a^ | Linear range  (μM) | R^2^ | LOD  (μM) | LOQ  (μM) | RSD% (n=5) | |
| --- | --- | --- | --- | --- | --- | --- | --- |
|  |  |  |  |  |  | Intra-day precision | Inter-day precision |
| DiAcSpm | y = 0.254x + 12.36 | 10-500 | 0.9984 | 3.3 | 9.8 | 4.1% | 3.8% |

^a^ where y is peak area (mV·min) and x is concentration (μM).
**Comment 18.** There are few pairs between this detection method and existing breast cancer screening methods. It is suggested to increase the comparison between this method and existing clinical methods to highlight the superiority and innovation of this method.

**Response:** Thank you very much for your constructive suggestions. We have added a section 3.7 on method comparison and the details added in **Table S2** in the **Supporting Information**. Please check the modification in the latest manuscript on **page 21, lines 432-445**.

**3.7 Method comparison**

As detailed in the comparative analysis (Table S2), the MIM-CME-HPLC-UV method established in this study exhibits distinct advantages for the determination of DiAcSpm. Notably, it eliminates the need for derivatization, thereby simplifying the sample preparation process relative to several existing approaches. Although the sensitivity (LOD of 3.3 μM) is lower than that of mass spectrometry-based methods, the proposed technique offers an excellent and wide linear range (10–500 μM), superior precision (both intra- and inter-day RSD < 4.1%), and highly consistent recovery rates. A key strength of this approach is the incorporation of a molecularly imprinted polymer (MIM) for selective extraction, which ensures high specificity and reusability (>95% capacity retention after 5 cycles). Combined with the cost-effectiveness and operational simplicity of the HPLC-UV platform, this method represents a robust, reliable, and practical alternative for the routine analysis of specific polyamines such as DiAcSpm in clinical samples.
**Comment 19.** It is suggested to supplement the research data on the service life of MIM column to improve the practicability of this method.

**Response:** Thank you very much for your constructive suggestions. We evaluated the reusability of the MIM column by performing 5 consecutive extraction-desorption cycles using a 200 μM DiAcSpm standard solution. The column retained >95% of its initial adsorption capacity after 5 cycles, with a relative standard deviation (RSD) of 3.8% for the extraction recovery. These results demonstrate excellent operational stability and reusability of the MIM column, making it suitable for repeated analyses in clinical sample processing. The data have been added to **Section 3.5** of the revised manuscript. Please find the results in **pages 18-19, lines 393-396：**The reusability of the MIM column was assessed over five consecutive extraction-desorption cycles, yielding an extraction recovery RSD of 3.8% and retaining >95% adsorption capacity, which demonstrates excellent operational stability for repeated clinical analyses.

**Comment 20.** In fact, the sensitivity is not well in the proposed method. Is the method suitable for medical diagnosis? Please provide the background knowledge and data for the target analyte detection, especially in clinic analysis.

**Response:** Thank you very much for your constructive suggestions. Reported demonstrate that urinary levels of N1,N12-diacetylspermine (DiAcSpm) and N1,N12-diacetylspermidine (DiAcSpd) were significantly elevated in breast cancer patients compared to healthy controls, with a substantial proportion of patients exhibiting values well above the established cut-off lines, particularly in those with advanced-stage disease. In terms of diagnostic performance, DiAcSpm emerged as the most promising marker, demonstrating a sensitivity of 46.4% for breast cancer detection, which was markedly superior to the sensitivity of conventional serum tumor markers carcinoembryonic antigen and CA 15-3. The study-defined cut-off values, reported in nmol per gram creatinine, were 298 nmol/g cre for DiAcSpm and 857 nmol/g cre for DiAcSpd. When estimated based on an average daily urine output of 1-2 liters in adults, these cut-off values correspond approximately to volumetric molar concentrations of 150-300 nM for DiAcSpm and 430-850 nM for DiAcSpd, collectively indicating that urinary DiAcSpm concentration represents a highly promising non-invasive tumor marker for breast cancer. This confirms that our method is sufficiently sensitive for clinical detection and monitoring. The above content has been added to the manuscript. Please check the modification in the latest manuscript on **page 3, lines 58-63**: Urinary N1,N12-diacetylspermine (DiAcSpm) is significantly elevated in breast cancer patients and possesses considerable potential as a non-invasive diagnostic biomarker, with an estimated cut-off of 150-850 nM and a sensitivity of 46.4% that surpasses conventional serum biomarkers, as measured by competitive ELISA (Enzyme-Linked Immunosorbent Assay) [12].

**Reference**

**[12]** Umemori Y, Ohe Y, Kuribayashi K, Tsuji N, Nishidate T, Kameshima H, Hirata K, Watanabe N (2010) Evaluating the utility of N1,N12-diacetylspermine and N1,N8-diacetylspermidine in urine as tumor markers for breast and colorectal cancers. *Clinica Chimica Acta* 411:1894-1899
 Based on the research, while the current analytical sensitivity of the developed MIM-CME-HPLC-UV method for DiAcSpm detection requires further improvement to meet clinical concentration thresholds, the study establishes a strong methodological foundation with several distinct advantages that underscore its potential value. The method demonstrates exceptional selectivity and precision, achieving relative standard deviations (RSD) of less than 4.1% for both inter- and intra-day measurements. This high reproducibility, facilitated by the hydrophilic amphiphilic molecularly imprinted polymer (MIM) column, is critical for ensuring reliable and accurate analyses. Furthermore, the MIM column exhibits remarkable operational stability and reusability, maintaining over 95% of its adsorption capacity and an extraction recovery RSD of only 3.8% across five consecutive extraction-desorption cycles. This robustness indicates excellent potential for cost-effective application in high-throughput settings.

Most importantly, the core methodology presents a highly promising and adaptable platform. The combination of molecularly imprinted solid-phase extraction with HPLC-UV detection provides a sound architectural basis. The primary advantage of this work lies not in its immediate performance but in its significant potential for future optimization. By focusing on enhancing sensitivity to bridge the gap with clinical needs—specifically, by improving the limit of detection to match the sub-micromolar range of urinary DiAcSpm—this methodological framework could be rapidly transformed into a highly competitive and clinically applicable assay. Thus, the study delivers a robust, precise, and stable technological foundation poised for impactful development.
